# Showcasing the contribution of social sciences to health policy and systems research

**DOI:** 10.1186/s12939-018-0862-5

**Published:** 2018-09-24

**Authors:** Stephanie M. Topp, Kerry Scott, Ana Lorena Ruano, Karen Daniels

**Affiliations:** 1 College of Public Health, Medical and Veterinary Sciences, James Cook University, Townsville, 4810 QLD Australia; 2 Nossal Institute for Global Health, University of Melbourne, Melbourne, 3002 VIC Australia; 3 Research Consultant, Bengaluru, Karnataka India; 4 Johns Hopkins Bloomberg School of Public Health, 615 North Wolfe Street, Baltimore, 21205 USA; 5 Center for the Study of Equity and Governance in Health Systems, Guatemala, Guatemala; 6 Center for International Health University of Bergen, Bergen, Norway; 7 Health Systems Research Unit, South African Medical Research Council, Cape Town, South Africa; 8 Health Policy and Systems Division, School of Public Health and Family Medicine, University of Cape Town, Cape Town, South Africa

**Keywords:** Health systems, Health policy, Social science, Qualitative, Equity

## Abstract

**Background:**

This Special Issue represents a critical response to the frequent silencing of qualitative social science research approaches in mainstream public health journals, particularly in those that inform the field of health policy and systems research (HPSR), and the study of equity in health.

**Methods:**

This collection of articles is presented by SHAPES, the thematic working group of Health Systems Global focused on social science approaches to research and engagement in health policy and systems. The issue aims to showcase how qualitative and theory-driven approaches can contribute to better promoting equity in health within the field of HPSR.

**Results:**

This issue builds on growing recognition of the complex social nature of health systems. The articles in this collection underscore the importance of employing methods that can uncover and help explain health system complexities by exploring the dynamic relationships and decision-making processes of the human actors within. Articles seek to highlight the contribution that qualitative, interpretivist, critical, emancipatory, and other relational methods have made to understanding health systems, health policies and health interventions from the perspective of those involved. By foregrounding actor perspectives, these methods allow us to explore the impact of vital but difficult-to-measure concepts such as power, culture and norms.

**Conclusion:**

This special issue aims to highlight the critical contribution of social science approaches. Through the application of qualitative methods and, in some cases, development of theory, the articles presented here build broader and deeper understanding of the way health systems function, and simultaneously inform a more people-centred approach to collective efforts to build and strengthen those systems.

## Editorial

This Special Issue represents a critical response to the frequent silencing of qualitative social science research approaches in mainstream public health journals, particularly in those that inform the field of health policy and systems research (HPSR), and the study of equity in health [1–3]. The issue is presented by SHAPES, a thematic working group of Health Systems Global (a membership-based society which aims to convene researchers, policymakers and implementers to develop the field of HSPR) focused on social science approaches.

By bringing together this collection of articles, the special issue highlights the critical contribution of qualitative social sciences including  interpretivist, critical, emancipatory, and other relational methods to our understanding of health systems, policies and interventions. Today, political, professional and disciplinary structures continue to privilege positivist research and quantitative methods, attributing greater evidential value to the knowledge produced by these approaches. This issue builds on growing recognition of the complex social nature of health systems [4] and on the understanding that utilizing *only* positivist research approaches in the study of health and health systems contributes to stripping away human experience and context. Articles in this issue demonstrate the importance of employing qualitative social science methods to explore the perspectives, experiences, relationships and decision-making processes of human actors within health systems, and in so doing, help uncover and explain the impact of vital but difficult-to-measure issues such as power, culture and norms. Through their application of qualitative methods and, in some cases, development of theory, they help build a broader and deeper understanding of the way various health systems function, and simultaneously inform a more people-centred approach to collective efforts to build and strengthen those systems.

Linked by two important themes, this initial collection of six research papers and two commentaries cut across a range of social science approaches and include policy analysis, rapid ethnography, and theory driven sociological enquiry. Future papers will be added to an online thematic collection on a rolling basis.

### Global policies, local realities

The ways in which global health policies are absorbed into national and subnational health systems, and their impact as they interact with local realities is a strong theme running through the issue.

Contractor, et al. [5] use rapid ethnography to explore the dissonance between tribal women’s perception of pregnancy and childbirth, and the Indian health system’s approach to maternity care in the context of a national policy that strongly incentivises facility-based birth. Drawing on five months of data collection in Odisha state, this exploratory study used qualitative methods to document how different actors perceived and experienced the policy. Unstructured group discussions explored community perceptions around pregnancy and childbirth; in-depth interviews explored women’s actual experiences and practices of pregnancy and childbirth; key informant interviews with service provides yielded contextual information about the field area and views from within the health system; and observations enabled triangulation and produced first-hand information about the location and conditions of health services and tribal areas. The authors highlight the tensions between priorities embedded within national-level policies and tribal women’s own preferences and needs when it comes to childbirth. Their narratives demonstrate how multiple financial, geographic, social and cultural factors mitigate against uptake of facility-based maternity services, and result in pressure, sometimes coercion, by local health system actors, to comply. The article demonstrates the importance of qualitative methods and grounded analysis for surfacing the unintended consequences of blanket state policies through documentation of its impacts on so-called beneficiaries.

Also focussing on India, Sriram, et al. [6] present a nuanced, contextually rich analysis, reflecting on the way that actors from high-income countries and members of the extended Indian diaspora contribute to socialisation and legitimation of a new medical speciality (emergency medicine). The research draws on a full year of qualitative data collection conducted by the first author including interviews with 76 participants across 11 towns/cities within India, review of 248 documents and observation of 6 meetings. The authors use framework analysis, applying concepts from the literature to insights emerging from the reading of the data, and brought both emic (subject; the first author is a member of the diaspora) and etic (observer) perspectives in making sense of the data. They point to the way power within these networks resulted in the rapid growth of the speciality of emergency medicine, but also influenced its evolution as a highly medicalised, tertiary-level form of care, inaccessible to the majority of Indians for structural reasons including affordability and availability. The authors note that the socialisation of domestic Indian stakeholders in this field ‘flows from a long history of LMIC (low- and middle-income country) stakeholders adopting ideas from high-income countries, driven by undercurrents of globalization and innovations in communication and technology’. Through the personal accounts of stakeholders with a range of perceptions and experiences in relation to the growth of emergency medicine, the authors interrogate and debunk the positive narrative of knowledge flow from high-income countries to LMICs. The qualitative analysis presented instead paints a complex picture, in which power influences knowledge transfer, the outcome of which is not always experienced as beneficial or positive.

Lodenstein, et al. [7] describe the contradictory role played by traditional leaders in Malawi in the pursuit of improved reproductive health outcomes. They bring attention to the power of traditional leaders, who are regarded as key to facilitating community adoption of positive public health norms including earlier and more frequent attendance at clinic-based antenatal visits. In recent times, the adoption of public health norms in Malawi has been driven by by-laws, set by traditional leaders and with often punitive consequences for those who do not comply; for example imposing fines on women who do not attend antenatal care or who are not accompanied by their husbands on those visits. While some have heralded the success of such by-laws, the authors use qualitative methods and a gendered perspective to explore these as a social process of norm formulation from the perspective of stakeholders involved in by-law creation, as well as the perspective of those affected by them. Recognising that norms are expressed in multiple ways (rules, behaviours, narratives and mechanisms of enforcement), the authors collected data from various sources (documents, observations, and interviews), so as to explore this range of expression. They show that although by-laws were meant to strengthen service uptake and improve health outcomes for pregnant women, they also resulted in the most vulnerable women bearing the moral and material responsibility for any perceived failure to meet reproductive health policy and targets. This study, which is grounded in rich contextual experience, provides important information to national and global health systems decision makers who may be considering using traditional lines of authority to enhance uptake of public health interventions.

### Resources and mechanisms of redress

While the above papers describe, and to differing extents deconstruct, the ways in which health systems interact with and exacerbate broader social and structural inequities, a second harmonizing theme in this collection is the way different resources and mechanisms can be mobilised as a form of redress to such inequities.

Spanning both themes showcased in this issue, Turcotte-Tremblay, et al. [8] describe the local effects of a globally touted performance-based financing (PBF) policy in Burkina Faso. The authors examine the equity measures (such as user fee exemptions available to those holding an indigent card) within PBF, which were introduced to address inequitable access. The study is framed using Rogers’ diffusion of innovations theory. In a comparative case study design across four primary health services, the authors utilise empirical methods, including 93 interviews, discussions, observation and document analysis. Using primary data the authors are able explore the way multiple local actors, including members of local indigent selection committees, re-invented elements of the PBF equity measures over which they had control, to either increase their relative advantage or to adapt to implementation challenges and context. For example, distributing free or very low-cost medications led to financial difficulties and drug shortages at some clinics and compensatory actions intended to resolve these problems by the ‘street level bureaucrats’ running front-line services led to adverse knock-on impacts for clients. Ultimately, the authors demonstrate how local knowledge of what it means to be indigent, and the power dynamics inherent within the health services, interacted with PBF implementation to result in both ‘uncertain and unequal’ coverage of the policy.

Topp, et al. [9] report on an empirical study of a policy-driven effort to improve the social accountability of prison health services in Zambia through the establishment of prison health committees. Locating their work in the discipline of public policy, the authors use a combination of interviews, focus groups and ethnographic observation, and begin by exploring Joshi’s three domains of impact for social accountability interventions: state responsiveness (represented by facility-based prison officials), societal impact (represented in this study by inmates), and state-society relations. (represented by relations between inmates and prison officials). Their analysis reflects on the ways in which power relations became less hierarchical, and how health outcomes improved in one particular prison after the introduction of a staff-inmate committee. A second phase of analysis draws on a more theoretical and (hence) more widely generalisable model comprising three intersecting ‘axes’ of accountability: power, ability and justice [10], using these axes to examine the depth and breadth of committee impact. The authors conclude that in relation to prison health care, local context as well as national level politics and legislative reforms, “will be crucial to support democratic decision-making, authentic engagement and appropriate action” in prison health services in low-income settings.

Kapilashrami and Marsden [11] report a study of access to health-enabling resources by multiply-disadvantaged groups in a deprived part of Scotland. Drawing on human geography and political science, their research uses the theoretical concept of intersectionality – that is, “the multiple interacting influences of social location, identity and historical oppression” – and a combination of standard qualitative tools (interviews, focus groups) and more contemporary and participatory methods (notably collaborative health resource mapping). The authors find that health-enabling resources were variously material, environmental, cultural or affective, with the combined influence of these resources playing out differently for different individuals. Amartya Sen observed in his health capabilities framework, the need to consider both individual choices and societal *chances* [12]. Through their use of multiple qualitative methods and the application of intersectionality, the authors demonstrate how individual responsibility, and blame, for health-related behaviour choices is an impoverished explanatory framework because it overlooks the institutional, structural and environmental influences on such behaviours.

While methodologically heterogenous, the articles in this issue showcase just some of the ways in which qualitative social science methods generate important new knowledge that is sensitive to context and which can act as a means to ‘un-silencing’ voices on the margins. Greenhalgh [13] in her commentary highlights these points, discussing the important role of *critical* social science as an underutilised method of social critique and emancipation of oppressed groups. She notes that methods such as these ask, “whose definitions count?”; “who makes the rules?”; and “whose voice is *not* being heard?”

In their commentary too, Lewin and Glenton [14] note that perhaps *the* key role of qualitative social sciences is to represent “the views and experiences of stakeholders, including vulnerable and marginalised groups who are often not represented directly.” And indeed, this collection represents a clear body of evidence that health policies and system levers require much work. Contractor, et al. [5], Kapilshrami, et al. [11] and Lodenstein, et al.’s [7] articles, in particular, demonstrate how qualitative social science research can surface issues experienced by, and present the voices of, people on the ground, helping to hold to account global and national health systems leaders responsible for health policy and planning. In order to realise the full value of this type of evidence, however, Lewin and Glenton [14] also argue the need for more investment in our collective capacity to synthesise the knowledge generated, and to work more closely with policy users and other stakeholders to build their capacity for evidence use.

Articles in this issue demonstrate how qualitative social science methods may be used to engage and participate with actors to co-produce knowledge, evidence and even solutions for change [1]. At inception, the idea for this special issue also encompassed ambitious plans to model a participatory and empowering approach through mentorship of early career authors, as well as for those based in LMICs. These ideas align with the values of people-centredness and equity that underpin the broader mission of Health Systems Global. Many of the lead authors in this issue are early career researchers, although most are either based in, or receive substantial support from institutions in high-income settings. We therefore believe more personal and institutional investment in learning opportunities through webinars, online teaching, and one-to-one mentoring needs to be made available. This has been recently modelled through various initiatives undertaken by HSG members, affiliates, and thematic working groups. We also acknowledge the ongoing challenges experienced specifically by health system actors who work in, or alongside, services to find the time or receive the guidance necessary to write about what they do. Questions that arose in the process of collating this issue, and which require more, and deeper examination include: how should rich (practitioner) experiences be documented? Should such documentation be acknowledged as a form of research? And if so, where does it belong in a saturated, but often siloed, publication world?

Critically engaging with issues of inclusion, voice and power is vital to building equitable and people-centred health systems and must be at the heart of the research processes that support these systems. As showcased in this special issue, robust qualitative social science research is ideally suited to understanding the social systems that generate or limit opportunities for equity in health, and that must be engaged with and transformed to build truly people-centred health systems.

## Abbreviations

HPSR: health policy and systems research
HSG: Health Systems Global
LMIC: Low- and Middle Income Countries
PBF: performance based financing
SHAPES: social science approaches for research and engagement in health policy & systems
